# Gambling and virtual reality: unraveling the illusion of near-misses effect

**DOI:** 10.3389/fpsyt.2024.1322631

**Published:** 2024-02-01

**Authors:** Alessandro Quaglieri, Alessandra Pizzo, Clarissa Cricenti, Ginevra Tagliaferri, Francesca Valeria Frisari, Jessica Burrai, Emanuela Mari, Giulia Lausi, Anna Maria Giannini, Pierpaolo Zivi

**Affiliations:** ^1^ Department of Psychology, Sapienza University of Rome, Rome, Italy; ^2^ Department of Psychology of Development and Socialization Processes, Sapienza University of Rome, Rome, Italy

**Keywords:** problem gambling, risk-taking behavior, gaming addiction, adolescence, decision making, slot machine

## Abstract

**Introduction:**

Studying gambling behavior is a crucial element in reducing the impact of problem gambling. Nevertheless, most current research is carried out in controlled laboratory settings rather than real-life situations, which raises concerns about how applicable the findings are in the broader context. Virtual reality (VR) has proven to be a valuable tool and has been utilized in various experimental scenarios. A limited number of studies have employed VR to investigate gambling behaviors, and few have explored them in an older adolescent context.

**Methods:**

This study examined the behavioral and physiological effects of gambling behavior, including problem gambling, gaming addiction, and risk-taking decision-making in a sample of 36 high-school students aged between 18 to 20 years using an *ad-hoc* constructed VR scenario designed to simulate a slot-machine platform.

**Results:**

The behavioral results highlighted that participants reporting more problem gambling were sensitive to near-misses: i.e., they bet more after near-misses than after losses. This result may reflect the false belief that gamblers, after near-misses, are closer to winning. Physiological data showed that participants exhibited heart rate deceleration during the anticipation of the outcome, which has been suggested to represent a marker of feedback anticipation processing and hyposensitivity to losses.

**Discussion:**

Overall, this study provides evidence for a new VR tool to assess gambling behaviors and new insights into gambling-related behavioral and physiological factors. Implications for the treatment of problem gambling are discussed.

## 1 Introduction

The popularity of gambling represents an enduring paradox for economists and psychologists because most gamblers are fully aware of the popular saying “the house always wins”. Therefore, from an economic perspective, gambling has a negative expected value, meaning that over many attempts, accumulating debt is practically inevitable. Despite this awareness, people extensively engage in these risky bets, providing valuable insights into the mechanisms of human irrationality and the propensity to accept and pursue bets that defy logic and rational decision-making (1). The allure of gambling may stem from different psychological factors, such as the thrill of uncertainty, the hope of a big win, or the social aspect of participating in gambling activities (2–4).

Gambling behaviour is maintained by erroneous beliefs and difficulties in decision-making processes (5). Distorted beliefs about gambling might lead problem gamblers to overestimate their chances of winning and, therefore, continue gambling behaviors (6, 7). Moreover, emotional factors often override the calculation of expected losses, leading individuals to make choices that appear irrational from a purely economic point of view (8), while, different environmental gambling characteristics, such as slot machine winnings that are commonly accompanied by flashing lights and loud noises, seem to encourage and sustain cognitive distortions (9).

### 1.1 Near-misses effect

Cognitive biases often occur in the case of near-misses; near-misses, also known as near-wins, occur when elements of a game “suggest” to gamblers that they have almost achieved a winning outcome (10, 11). Near-misses are particularly prevalent in gambling games where the outcome is a random event, such as slot machines. Near-misses appear to be hedonically pleasurable as they provide visual aspects similar to those of winnings. The literature suggests that near-misses play a key role in addictive behaviour by leveraging learning processes (i.e., conditioned reinforcement) that appear to increase the propensity to continue gambling (12). Indeed, near-misses are perceived as more discouraging compared to complete losses (13–15), whereas gamblers report an increased motivation to continue betting (16). Different studies manipulated the frequency of near-misses, showing that about 30% of near-misses increased gambling behavior by promoting higher winning expectancy during slot machine simulations (17, 18).

In addition, neuroimaging studies have shown that near-miss outcomes involve reward brain circuits overlapping with the neural activity involved in monetary wins by involving a circuit of areas (i.e., ventral striatum and rostral anterior cingulate cortex) linked to reinforcement processing (13, 19) and that these responses may be enhanced in problem gamblers (20, 21). Moreover, especially in those gamblers who showed a tendency to “chasing losses”, the near-misses increased the desire to continue playing (21, 22), suggesting that “near-misses” can enhance the motivation of gamblers to pursue losses and continue playing, fueling the illusion of control, and increasing the motivation to keep playing to avoid losses (23, 24). Chasing losses appears to be strongly correlated with characteristics related to gambler’s impulsivity which makes gamblers more inclined to make irrational bets or pursue short-term gains without fully considering the long-term risks (25, 26). The ability to inhibit impulsive responses is closely related to self-regulation and impulse control; in problem gamblers response inhibition may be impaired, leading them to impulsively respond to gambling opportunities without assessing the potential negative consequences (27–29).

Gambling is often associated with strong emotional reactions, such as the euphoria experienced after a win or the frustration and anger after a loss. These emotions can influence decision-making processes and lead to compulsive gambling behaviors (22). The positive emotions associated with both wins and near-misses (i.e., the positive sensation of hype produced by a near-win) act as reinforcers, driving gamblers to bet to replicate the thrill of winning, even if they may incur significant losses. Gambling may also alleviate negative affect states such as boredom, anxiety, or low mood (i.e., negative reinforcement); these mechanisms of emotional learning play a key role in the development and maintenance of gambling behavior (30).

### 1.2 Physiological aspects of gambling

Gambling is also associated with physiological arousal that is manifested by increased heart rate and elevated cortisol levels (31–33). Gamblers’ arousal can be detected using specific physiological measures, such as Heart Rate Variability (HRV) and Galvanic Skin Responses (GSR) that are commonly used to monitor the body’s responses to various stimuli and events. Different studies have shown that a significant increase in HRV is observed during gambling compared to baseline and that it is therefore not the gambling itself that is significantly arousing, but it is the outcome, whether winning or losing, that influences HRV (34–36). By these findings, the study by Lole et al., (37) showed that wins produced larger SCRs than losses, specifically big wins rather than small wins. Electrodermal measures were also correlated with arousal ratings during gambling, as HR showed a slight deceleration before the outcome of the event, and HR rebound was greater after wins than after losses.

According to Griffiths (38); Griffiths (39), the ability of wins and near-misses to elicit physiological arousal means that gambler believes “they are not constantly losing but constantly nearly winning”. In this view, it would appear that near-misses have been shown to elicit win-like responses, such as increased HR and motivation and persistence in gambling behaviors. Specifically, Hultman et al., (40) found that two types of near-misses, in which the last winning symbol is one position before or after the pay line, seem to generate different physiological responses in both HRV and SCRs. While near-misses in which the reel stops one position before the pay line are perceived as more pleasant and are associated with increased motivation to play, compared to the other condition in which near-misses are perceived as aversive and demotivating (15, 21). An interesting insight showed that near-misses seem to elicit higher heart rate deceleration and increased SCRs compared to both wins or losses, probably due to the frustration related to a missing big win (41). Similar results are found by Lole et al., (42) concerning wins, losses, and near-wins, where both wins, and near-wins outcomes showed a greater physiological arousal than losses.

### 1.3 Virtual Reality in gambling research

Virtual reality (VR) enables the creation of ecologically valid scenarios and standardized delivery systems by combining computer graphics and peripheral devices (43), which provide opportunities to create and apply complex stimuli. This extends traditional methods of stimulus exposure by providing stimulus administration and real-time evaluations that incorporate inanimate and animate cues (44). Improved accessibility and exposure are some of the key features that VR can offer to create a more accessible and controlled environment for studying gambling behavior, having the advantage of being able to expose individuals to fully controlled and accessible social situations in a safe clinical environment to assess cognition, behaviors, emotions, and physiological responses in real-time (45).

VR can be a useful tool in gambling studies, both for prevention and intervention in gambling disorders: VR can be used to create realistic simulations that can be used in Exposure Therapy or Cognitive-Behavioral Intervention, through a controlled environment without spending real money, making it a safer space (46). Immersion in VR can evoke the desire and positive expectation of gambling and is also effective in identifying high-risk situations and dysfunctional thoughts.

VR has been used as a tool to study the effects of near-misses in various contexts, including gambling, gaming (47), and other activities (48) involving risk and chance. Within gambling and gaming, near-misses can be particularly significant because they can trigger feelings of excitement and anticipation, leading individuals to believe they are close to a win and encouraging them to continue playing or gambling (49). Participants often report increased excitement and arousal when encountering near-misses in VR simulations, similar to what is observed in actual gambling settings.

Near-misses in VR can lead to increased motivation and persistence in continuing the activity, in the hopes of achieving a win, replicating the real-world effect observed in gambling. Near-misses can contribute to cognitive distortions, such as the illusion of control or the belief in the “almost-won” phenomenon (50, 51). A recent study by Detez et al. (50) used immersive VR to study changes in physiological arousal and gambling behavior induced by near-misses: the authors found a significant acceleration of heart rate for both near-misses and losses compared to wins, indicating an initial orienting response. Both types of loss were associated with faster responses to the next spin, thus seemingly encouraging gambling, as participants experienced a more immediate acceleration of heart rate (indicative of excitement typical of losses) and triggered quicker responses.

Overall, the combination of VR and the study of near-misses provides researchers and clinicians with a valuable tool to better understand the psychological and behavioral aspects of gambling behaviors. By gaining insights into how near-misses affect individuals in controlled virtual environments, researchers can develop more targeted interventions for problem gambling and related addictive behaviors.

### 1.4 Gambling among older adolescents

Older adolescence (15-19 years according to the World Health Organization WHO) is a highly sensitive developmental window for the emergence of gambling-related problems (52, 53). In fact, during adolescence, there is a possibility of an escalation in patterns of engagement with various forms of gambling and gambling-like activities (54–56). The evolution of gambling and video game opportunities has increased the chances for young people to engage in gambling through different platforms, technological devices, and gaming venues (54, 57–59). Exposure to these behavior-reinforcing technologies is potentially correlated with an increased risk of problematic gambling (60, 61).

In particular, several studies have established positive associations between increased video game frequency and problematic gambling (60, 62, 63). Beyond this association, Internet gaming disorder (IGD) has been proposed as a behavioral addiction, like gambling disorder, in the section recommending conditions for further research of the DSM-5 (64) for different age groups (62, 63, 65, 66). Furthermore, gaming disorder has been defined by the WHO as a “pattern of persistent or recurrent gaming behavior” and was included in the 2018 release of the 11th revision of the International Classification of Diseases (ICD-11) (67, 68).

It is known that many adults who have experienced gambling problems often reported having started gambling before the age of 18 (69, 70) and excessive gambling in adolescence can often be an indicator of other underlying problems associated with a developmental period (69).

In addition, problematic gambling has intergenerational influences; individuals whose parents have had gambling or other addiction problems are more likely to develop similar problems themselves (71). Although this influence may be supported by genetic and neurobiological factors that make individuals vulnerable to addictive behaviors (e.g., poor impulse control, attention problems), there are also social and cultural factors involved (e.g., families that approve and normalize gambling; (72).

While the impact of gambling on the health of adults is increasingly recognized, few studies have focused on replicating these results among adolescents. Consistent with studies on adults, Cardwell et al. (73) found that adolescent gamblers suffered greater health problems (i.e., anxiety, stress) and worse functioning (i.e., poor school performance, greater use of alcohol and illicit drugs, greater likelihood of endorsing violence-related behaviors, associated with maladaptive emotion regulation styles). Other studies (74–77) show that gambling in adolescents is also associated with various mental health issues, such as high levels of impulsivity, anxiety, depression, and stress.

### 1.5 Aims and hypotheses

The present study aimed to measure outcome-related physiological and behavioral responses to test the reliability and validity of a simplified VR-based slot machine task. Furthermore, we considered gaming addiction, as it might play a role in the VR experience of older adolescents. As a secondary aim, we included gambling-related (i.e., gambling motives, cognitions, and internet gaming), emotion-related (i.e., negative affect, regulation, dysregulation, and alexithymia), and personality (i.e., impulsivity and sensation-seeking) subjective measures to investigate which of the self-reported measures considered can predict gambling severity. The self-reported measures were selected according to the previous literature showing different risk factors and risk markers of gambling disorder (78). The gambling severity reflects the extent to which participants present a degree of risk of problem gambling measured by the South Oaks Gambling Screen for Adolescents (SOGS-RA).

At the behavioral level, we measured risky behavior and hypothesized:
 **H1**: we expect that participants make more risky bets after a near-miss outcome, especially for problematic gamblers, compared to following wins and losses.
 
**H2**: we expect higher decision times following wins than following near-misses and losses, reflecting post-reinforcement pauses (12), and lower decision times following near-misses than following losses, which may reflect a desire to alleviate the frustration from nearly-winning quickly.
At the physiological level, we hypothesized that:
 
**H3:** we expect a greater GSR response to winning outcomes and a greater response to near-miss than loss outcomes.
 
**H4:** we expect lower inter-beat-intervals (IBI) before the feedback presentation that could represent a marker of feedback anticipation and processing, regardless of the type of outcome.

## 2 Methods

### 2.1 Participants

Only participants above the legal age of 18 were recruited from different upper secondary schools. Exclusion criteria for the research included the presence of psychotic spectrum disorders, progressive neurodegenerative disorders, and neurological disorders. Participation in this study was voluntarily. The study was conducted in accordance with the Declaration of Helsinki and was approved by the Institutional Review Board of the Department of Psychology, “Sapienza” University of Rome with protocol number (273/2022), and all participants provided informed consent. A sensitivity analysis using MorePower 6.0 software showed that with a total number of 36 participants with a power of 0.80, we were able to detect an effect size of.099 partial eta squared. The final sample consisted of 36 participants (22 males) aged between 18 and 20 years (M = 18.17; SD = 0.50). Participants who had partially completed the research protocol were excluded. All participants were students and attended high school (N = 36, 100%). Concerning screening measures investigating the presence of behavioral addiction of interest (i.e., SOGS-RA and IGD), the sample reported mean scores of 1.86 (SD = 2.05) and 14.72 (SD = 7.16) for gambling severity and internet gaming disorder, respectively.

### 2.2 Procedure

Before the experiment, participants were briefed about procedures and provided informed consent followed by a brief description of the study and the functionality of the head-mounted display, and potential symptoms of cybersickness. The participants recruited in the study already completed questionnaires on the variables of interest (e.g., gambling severity, gambling distortion, emotion regulation, impulsivity) via the Qualtrics platform. Before entering in the VR scenario participants were equipped with the Galvanic Skin Response (GSR) and Heart Rate electrodes (HR), and 5 min of resting state data were acquired before the instructions for the slot machine task were read to the participant.

### 2.3 Questionnaires

The South Oaks Gambling Screen (SOGS-RA, Italian version, 79) is a self-reported 12-item questionnaire measuring several aspects related to gambling disorder such as loss of control, recovery from monetary losses, interference with family, school, and relational life, guilt feelings, and consequences of gambling. Have been involved in a gambling activity at least once in the previous year “defined” participants as “gamblers”. The SOGS-RA scale identifies three types of gamblers: not-problem (SOGS-RA score = 0-1); at-risk (SOGS-RA = 2-3); and problem (SOGS-RA score higher than 4). Students who reported having no experience of gambling in the previous year were defined as “not gamblers”. In this study, the Italian version of the SOGS-RA was used and reported to have a Cronbach’s alpha of. 91.

The Internet Gaming Disorder Scale - Short-Form (IGDS9-SF, Italian version, 80) is a self-reported 9-item questionnaire corresponding to the nine core criteria defined by the DSM-5 (64). This scale assesses the severity of IGD and its detrimental effects by examining both online and/or offline gaming activities occurring over 12 months. The participants are asked to respond on a 5-point Likert scale ranging from 1 (never) to 5 (very often). Higher scores are indicative of higher degrees of Internet Gaming Disorder. The IGDS9-SF showed an internal consistency coefficient of. 93.

The Sensation Seeking Scale - Brief version (BSSS, Italian version, 81) contains eight Likert-type items rated on a 5-point scale (from strongly disagree to strongly agree), yielding a maximum score of 40. It retains Zuckerman (82); Zuckerman (83) conceptualization that sensation seeking as a personality trait is composed of four components, namely thrill and adventure seeking, experience seeking, disinhibition, and boredom susceptibility. In this sample Cronbach’s alpha was. 66.

The Gambling Related Cognitions Scale (GRCS-I, Italian version, 84) is a self-reported 23-item scale to measure gambling-related cognitions and based on a 7-point Likert scale ranging from 1 (strongly disagree) to 7 (strongly agree). This scale assesses five biases of gambling: Gambling expectancies (GRCS-GE), Illusion of control (GRCS-IC), Predictive control (GRCS-PC), Inability to stop gambling (GRCS-IS), and Interpretative bias (GRCS-IB). The Cronbach’s alpha coefficient for the GRCS total score was. 96.

The Gambling Motives Questionnaire (GMQ-19, original version, 85) is a self-reported 19-item scale to measure gambling-related motives based on a 4-point Likert scale ranging from 1 (never or almost never) to 4 (almost always or always). The scale measures four main gambling motives: coping (e.g., “because it helps when you are feeling nervous or depressed”), enhancement (e.g., “because it’s exciting”), social (“because it makes a social gathering more enjoyable”) and financial (e.g., “because you enjoy thinking about what you would do if you won a jackpot”). The Cronbach’s alpha coefficient for the GMQ was. 96.

The Barratt Impulsiveness Scale (BIS-11, Italian version, 86), is a self-reported 30-item questionnaire developed in order to assess the personality and behavioral dimension of impulsiveness describing both impulsive and not-impulsive behaviors. The Italian version developed by Fossati et al., (86), in this sample results, showed a good fit index with the original factor structure with a Cronbach’s alpha of. 84.

The Depressive Anxiety Stress Scale (DASS-21, Italian version, 87) is a self-reported 21-item questionnaire based on a four-point rating scale (i.e., ranging from 0 = “did not apply to me at all” to 3 = “applied to me very much, or most of the time). The questionnaire was developed to assess three constructs: anxiety, depression, and stress. The higher the score, the more severe the emotional distress was. Excellent levels of reliability were detected in this sample. 92.

The Toronto Alexithymia Scale (TAS-20, Italian version, 88) is one of the most common measures of alexithymia, a multifaceted personality construct that represents a deficit in the cognitive processing of emotion. Participants are encouraged to evaluate twenty items on a 5-point Likert scale ranging from 1 (strongly disagree) to 5 (strongly agree). Higher scores on the TAS-20 indicate greater difficulties in the cognitive processing of emotion. The TAS-20 results in a total score and three subscale scores (i.e., Difficulty Identifying Feelings, Difficulty Describing Feelings, and Externally Oriented Thinking). In this study, we used the Italian version of the TAS-20 (88), in this sample results showed a good internal consistency with a Cronbach’s alpha of. 76.

The Difficulties in Emotion Regulation Scale (DERS-20, 89), is a self-reported questionnaire that assesses five dimensions of emotion regulation (i.e., Clarity, Not-acceptance, Awareness, Impulse, and Goals). In the present study, the Italian 20-item short-form version developed by Lausi et al. (89) was used. Items are rated on a scale of 1 (almost never) to 5 (almost always). Higher scores indicate severe difficulty in emotion regulation. The Cronbach’s alpha coefficient for the DERS-20 in this sample was. 87.

The Emotion Regulation Questionnaire (ERQ-10, Italian version, 90), is a self-reported 10-item questionnaire that consists of two dimensions: Cognitive Reappraisal and Expressive Suppression. These two strategies for regulating emotion are known to be crucial in the management of emotions. In this study, we used the Italian version developed by Balzarotti et al. (90), in this sample results showed good internal consistency with a Cronbach’s alpha of. 72.

### 2.4 VR Gambling Scenario

**Figure 1:**
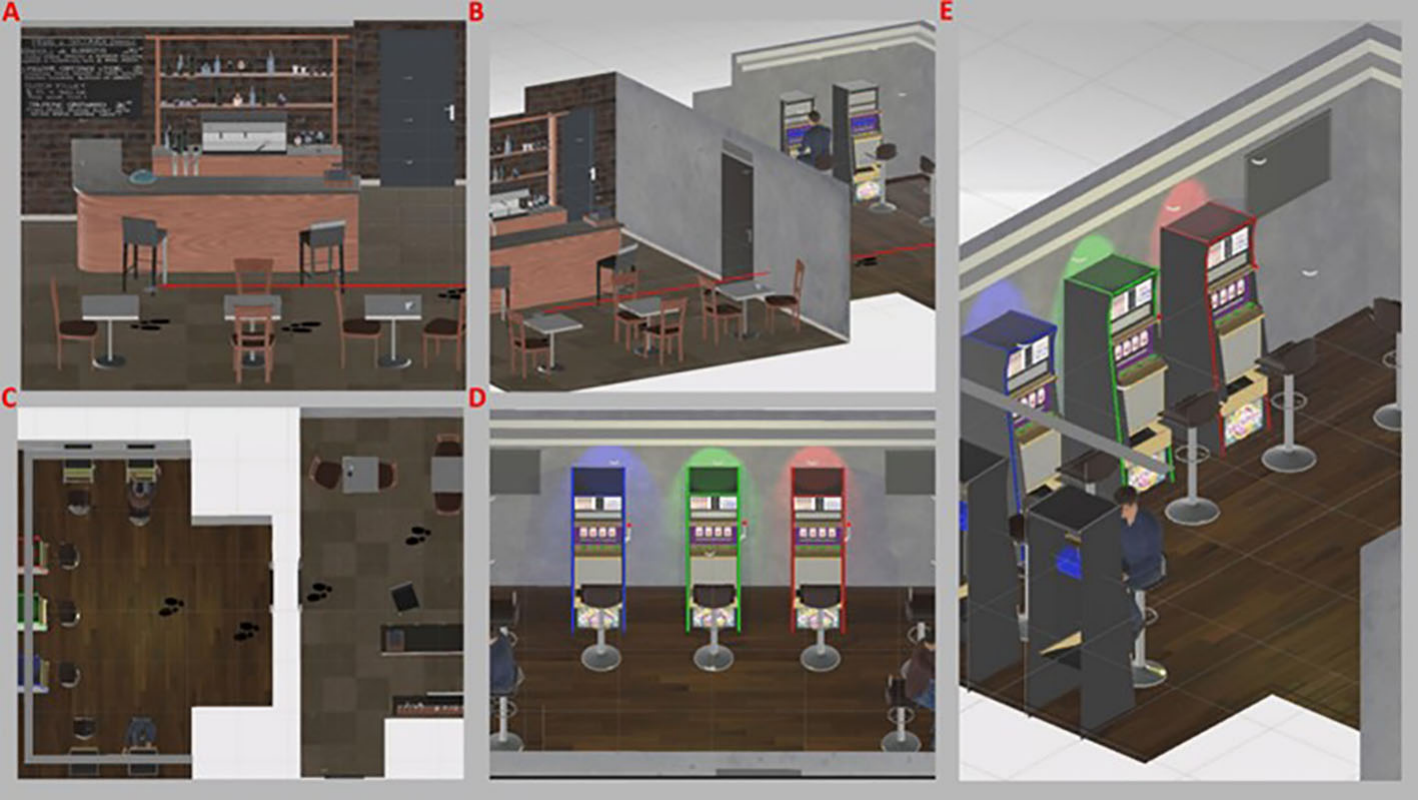
Virtual reality gambling scenario. **(A)** neutral “BAR-like” room; **(B)** side view of gambling room; **(C)** top view and gambling path; **(D)** experimental "slot-machine-like" room; **(E)** side view of experimental room.

The VR gambling scenario was developed on Windows-10 64-bit (Microsoft, Washington, United States) by using the Unity 3D engine (Unity Technologies, San Francisco, United States), the Oculus Utilities (v1.3.2), and the 3DS-Max 2014 (Autodesk, California, United States) for modeling 3D-objects. Scripted audio was integrated where appropriate. The scenario describes the arrival of a participant in a “Bar-like” environment, then through a predetermined path participants had to move into the second room (i.e., the slot-machine room) by using the one-handed joystick and footprints placed on the floor (**Figures 1A–D**). The participant autonomously selected one of the three available slot machines, which differed only in some visual characteristics (e.g., color, lights), but all three were programmed with the same pre-determined trial list and displayed an identical pay-line with a 4-reel design (**Figure 1**). A typical game round (approximate duration of 20 min) typically consists of: the selection of the bet amount (i.e., the number of points wagered), the start of the round (i.e., by pulling down the handle), and the payment of a potential win. Participants started with 2000 virtual points and could bet between 5, 10, and 25 points. Each trial started with a selection phase, during which participants selected the bet amount without time constraints. Afterward, participants had to pull the handle to start the spinning reel, which decelerated to a standstill in 6.0 s (anticipation interval). Each type of outcome was followed by a short melody (e.g., money or a neutral clip) and a message on the screen that read “You won”, “You lose”, or “Play again”, depending on the type of outcome. After the outcome was provided, participants were required to place a new bet.

**Figure 2:**
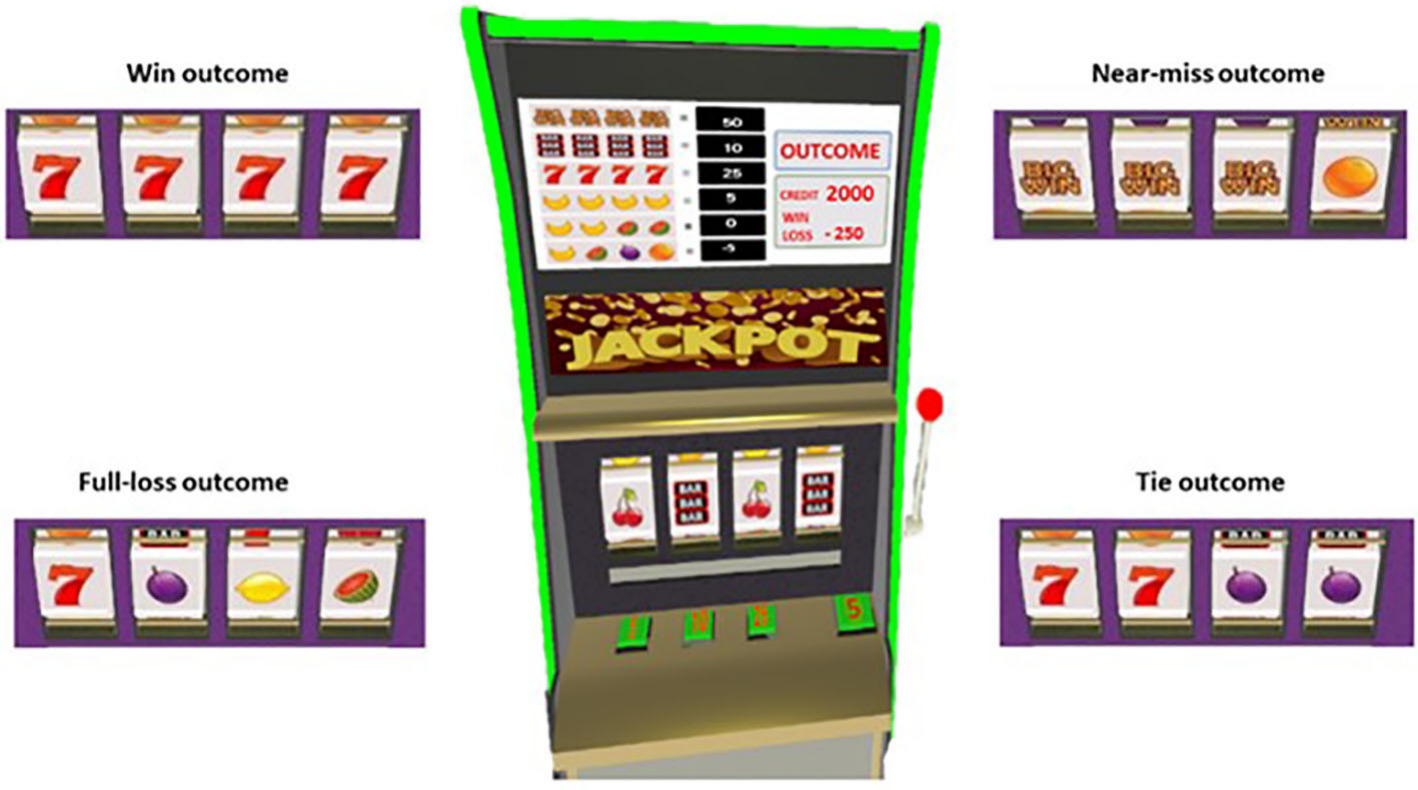
Slot-machine outcomes.

Wins occurred when all four reels showed the same icon, while losses occurred when the reel combinations consisted of four different icons. Ties occurred when there were two pairs of the same icons, and near-misses occurred when the first three symbols were the same while the fourth was different, but the winning symbol was in a position before the pay line (**Figure 2**). Based on the study by Hultman et al. (40), in which authors showed a different effect of two types of near-misses (i.e., before and after payline), we decided to implement only one type of near-miss (i.e., before the payline) that have been specifically associated with higher heart rate deceleration, higher subjective states of motivations, and slightly higher affective responses.

We presented four types of winnings (i.e., BigWin, BarWin, SevenWin, and FruitWin) with different icons and pay-out proportions (i.e., BigWin = X10; SevenWin = X5; BarWin = X2; FruitWin = X1). The participant could consult a legend, displayed at the top of the slot machine, which is updated according to the amount wagered in each round. The “Full-Loss” and “Near-miss” both involved the same monetary loss (i.e., the subtraction of the number of points wagered), while the “Tie” outcome involved no loss or win. The trial list of wins, losses, and ties was predetermined as 14 wins, 44 losses, 28 near-misses, and 15 ties for a total number of 101 trials. Our near-miss ratio (28%) was in line with the ideal percentage of near-misses as suggested by the literature (11, 13). We preferred to increase the occurrence of more potentially rewarding outcomes (i.e., N = 12 Almost BigWin, N = 9 Almost SevenWin, N = 7 Almost BarWin) to raise the chances of eliciting the effect of Near-Misses.

The first trial (i.e., the first tie) was removed from behavioral analyses. One participant re-started the VR scenario after performing 15 trials (i.e., due to a technical problem), and we included those trials in the calculation of measures for that participant.

### 2.5 Physiological measures

Heart Rate Variability (HRV) and GSR were recorded using the BIP2AUX and the GSR Sensor Unit adapters for the actiChamp-64 Plus amplifier at a sampling rate of 1000 Hz. Both signals were acquired in BrainVision Recorder and processed using BrainVision Analyzer.

#### 2.5.1 To record HRV

Three disposable pre-gelled Ag/AgCl electrodes were used and positioned by the Einthoven’s triangle superimposed on a human thorax (i.e., just below the clavicles). Specifically, the lead I (i.e., the positive electrode) was attached to the left arm (LA), the lead II (i.e., the negative electrode) was attached to the right arm (RA), and the ground electrode was used as a reference and placed on the lower left costal margin. After the ECG recording, data were pre-processed to remove noise due to subjects’ movement, breathing, and muscle electrical activity. A high-pass filtering with a 0.1 Hz cutoff frequency was applied to remove baseline wander, low-pass filtering with a 15 Hz cutoff frequency was used to filter electromyogram artifacts, and a notch filter was applied to remove 50 Hz power line interference. Then, all RR intervals on the ECG were detected using the Cardioballistic artifact correction (i.e., as implemented in BrainVision Analyzer). The ECG signals were re-sampled at a frequency of 256 Hz. For each outcome (near-misses, wins, losses, and ties), inter-beat-intervals (IBIs) were calculated as the temporal distance between R-waves of each pair of consecutive heartbeats (41). We calculated the six IBIs for the seven heartbeats immediately preceding the stop of the last reel to inspect the outcome-related heart-rate deceleration trends (IBI 6 refers to the temporal distance between the last two heartbeats before the stop of the last reel; IBI 5 refers to the interval between the penultimate and the third-to-last, and so on). With this strategy, we were able to capture changes in inter-beat intervals as the consecutive reels stopped before the outcome being revealed. To capture the whole heart-rate deceleration trend preceding the final stop, IBIs were calculated only between the heartbeats following the handle’s pull. Therefore, the number of data points for IBI 1 was slightly numerically lower than for IBI 6 in participants exhibiting a slow heart rate.

#### 2.5.2 To record the GSR

Two disposable isotonic electrolyte pre-gelled electrodes were used and positioned on the ventral medial phalanx of the index and middle fingers of the participant’s non-dominant hand. A high-pass filter with a 0.05 Hz cutoff frequency and a low-pass filter with a 5 Hz cutoff frequency were applied to the raw signal, Data were down-sampled at 250 Hz. The SCR (Skin Conductance Response) magnitude was analyzed to provide useful information about levels of arousal related to slot-machine outcomes (91). The SCR magnitude was calculated by considering a three-second time window starting one second after stopping the last reel (92), as the difference between the maximum value within the window and the value at the beginning of the window. Only positive differences were retained for data analysis and a square-root transformation was applied on them.

For both IBIs and SCR magnitudes, a recursive outliers removal procedure as in Van Selst and Jolicoeur (93) was applied, to account for the unbalanced number of trials among the four outcome conditions (from 0 to a max of 4.9% of the IBIs and a max of 5.7% of the SCRs were considered outliers and excluded). Due to technical issues, physiological data from two participants were not analyzed.

## 3 Statistical analysis

Statistical analyses were performed by using SPSS software (version 26). First, the internal consistency of the subjective scales was evaluated using Cronbach’s Alpha; the results showed high internal consistency with an alpha ranging from.66 to.96. For each variable, the assumption of normality was tested by analyzing the skewness and kurtosis indices, and a log-transformation was applied to highly non-normal scores (i.e., Social Motives of GMQ-19; Gambling Expectancies; Illusion of control; Inability to stop Gambling of GRCS-I). Differences between groups were tested with a multivariate analysis of variance, and in the pairwise comparisons, a Bonferroni correction was applied.

To study participants’ risk-taking, we analyzed the participants’ betting choice (i.e., the bet magnitude) following each slot machine outcome, considering the 5-coin bet as a “safe” bet and the 25-coin bet as a “risky” bet. We considered only 5 and 25-coin bets and excluded from the final analyses the 10-coin bet to consider bets reflecting more the “risk” and “safe” conditions since they are “the “minimum” and the “maximum” that participants could bet. Therefore, we calculated a risky bet ratio (e.g., Number of risky choices/Number of risky choices + Number of safe choices) after each slot-machine outcome. We also analyzed participants’ decision time after each outcome.

With respect to the VR task, we conducted a 2x4 ANCOVA with Outcomes (i.e., Win, Loss, Near-Miss, Tie) as a within-subjects factor for both the participant’s bet amount (i.e., BET) choice and decision time (DT). According to the SOGS-RA score, a between-subjects factor was also included by splitting participants into not-problematic (score of 0 or 1, N = 21) and problematic gamblers (2 or more, N = 15). The Gaming Addiction score was included as a continuous covariate (mean value of 14.72) to control for possible differences in the sample that may play a role in the VR experience. The 19% (N = 7) of the participants fulfilled the criteria for the IGDS-9 score for IGD diagnosis according to the Italian validation of IGDS-9 by 80.

Concerning the DT analysis, the covariate did not reveal any statistically significant interaction or main effect with a p-value >.05 and it was consequently removed from the final analysis. For decision times we excluded values higher and lower than 3 SDs from the participants’ mean (94).

With respect to the HRV, data were analyzed by using a 2x4x6 mixed ANCOVA with Group (i.e., not-problematic and problematic gamblers) as a between-subjects factor, Outcomes (i.e., Win, Near-miss, Loss, and Tie) and IBI (i.e., IBI-1, IBI-2, IBI-3, IBI-4, IBI-5, IBI-6) as within-subjects factors, and gaming addiction as a covariate. The covariate did not reveal any statistically significant interaction or main effect with p-values >.05 and it was consequently removed from the final analysis. With respect to the GSR, we used a 2x4 mixed ANCOVA with Outcomes (i.e., Win, Near-miss, Loss, and Tie), as a within-subjects factor and Group (i.e., not-problematic, and problematic gamblers) as a between-subjects factor and gaming addiction as a covariate. Even in this case, the covariate did not reveal any statistically significant interaction or main effect with a p-value>.05 and it was consequently removed from the final analysis. For all the ANOVAs, the Greenhouse-Geisser correction was applied in case of significant violation of sphericity.

To investigate which variables ultimately influenced the SOGS score, we performed a linear regression; in this procedure, we included stepwise all self-reported measures (i.e., DASS-21, BIS-11, ERQ-10, DERS-20, TAS-20, GMQ-19, GRCS-I, BSSS) and for behavioral risky choices variables. To reduce the number of variables entered, we included in the regression analysis total scores, except for the ERQ-10 and GMQ-19 for which we have included subdimensions. The variables included in the final model (i.e., according to criteria: probability-of-F-to-enter <= .050; probability-of-F-to-remove >= .100) entered in a separate linear regression model with bootstrapped (i.e., N = 1000) confidence intervals. Statistical significance was defined as p <.05.

## 4 Results

According to the SOGS-RA score, of the 36 participants (N = 21, 58.33%) were classified as not-problematic gamblers (SOGS-RA < 2) and (N = 15, 41.67%) were classified as problematic gamblers (SOGS-RA > 2) and constituted the two groups included in the analyses for this study (79). With respect to the self-reported measures there were statistically significant differences between groups in IGDS-9 (p <.05), BISS-11-Tot (p <.01), GMQ-F (p <.01), GMQ-C (p <.05) and GRCS-Tot (p <.01), in which problematic gamblers group scored higher than not-problematic gamblers group. All other self-reported measures did not statistically differ between groups; all differences between groups were reported in **Table 1**.

**Table 1:** Differences between groups for all self-reported measures.

	F	df	p	ή_2_	Group	N	Mean	SE
Age	1.00	1,34	>.05	–	Not-problematic gamblers	21	18.10	0.11
					Problematic gamblers	15	18.27	.013
IGDS-9	6.19	1,34	<.05*	0.15	Not-problematic gamblers	21	12.38	1.46
					Problematic gamblers	15	18.00	1.73
DASS-Tot	.304	1,34	>.05	–	Not-problematic gamblers	21	15.43	2.30
					Problematic gamblers	15	13.47	2.72
BIS-11-Tot	8.37	1,34	<.01**	0.19	Not-problematic gamblers	21	57.71	2.27
					Problematic. gamblers	15	67.87	2.68
ERQ-S	.040	1,34	>.05	–	Not-problematic gamblers	21	14.81	1.05
					Problematic. gamblers	15	15.13	1.24
ERQ-R	.391	1,34	>.05	–	Not-problematic gamblers	21	26.48	1.73
					Problematic. gamblers	15	24.80	2.05
DERS-Tot	.675	1,34	>.05	–	Not-problematic gamblers	21	47.57	2.90
					Problematic. gamblers	15	51.27	3.44
TAS-Tot	2.35	1,34	>.05	–	Not-problematic gamblers	21	53.19	2.36
					Problematic. gamblers	15	58.80	2.79
GMQ-S	3.52	1,34	>.05	–	Not-problematic gamblers	21	6.29	0.75
					Problematic. gamblers	15	8.47	0.89
GMQ.F	8.15	1,34	<.01**	0.19	Not-problematic gamblers	21	4.91	0.58
					Problematic. gamblers	15	7.47	0.69
GMQ-C	5.44	1,34	<.05*	0.13	Not-problematic gamblers	21	0.73	0.03
					Problematic. gamblers	15	0.83	0.03
GMQ-E	4.05	1,34	>.05	–	Not-problematic gamblers	21	0.77	0.03
					Problematic. gamblers	15	0.88	0.04
GRCS_Tot	13.07	1,34	<.01**	0.27	Not-problematic gamblers	21	28.67	4.05
					Problematic. gamblers	15	51.33	4.79
BSSS_Tot	0.99	1,34	>.05	–	Not-problematic gamblers	21	26.00	1.08
					Problematic. gamblers	15	27.67	1.28

*p <.05; **p <.01; IGDS-9, Internet Gaming Disorder; SOGS, South Oaks Gambling Severity; DASS-Tot, Depression,Anxiety, Stress Scale; BIS-11-Tot, Barratt Impulsivity Scale; ERQ-S, Emotional Regulation Scale Suppression; ERQ-R, Emotional Regulation Scale Reappraisal; DERS-Tot, Difficulties in Emotional Regulation Scale; TAS-Tot, Toronto Alexithymia Scale; GMQ-S, Gambling Motives Questionnaire; GMQ-F, Gambling Motives Financial; GMQ-C, Gambling Motives Coping; GMQ-E, Gambling Motives Enhancement; GRCS-Tot, Gambling Related Cognition Scale; BSSS-Tot, Sensation Seeking Scale - Brief version.

### 4.1 Behavioral results

**Figure 3:**
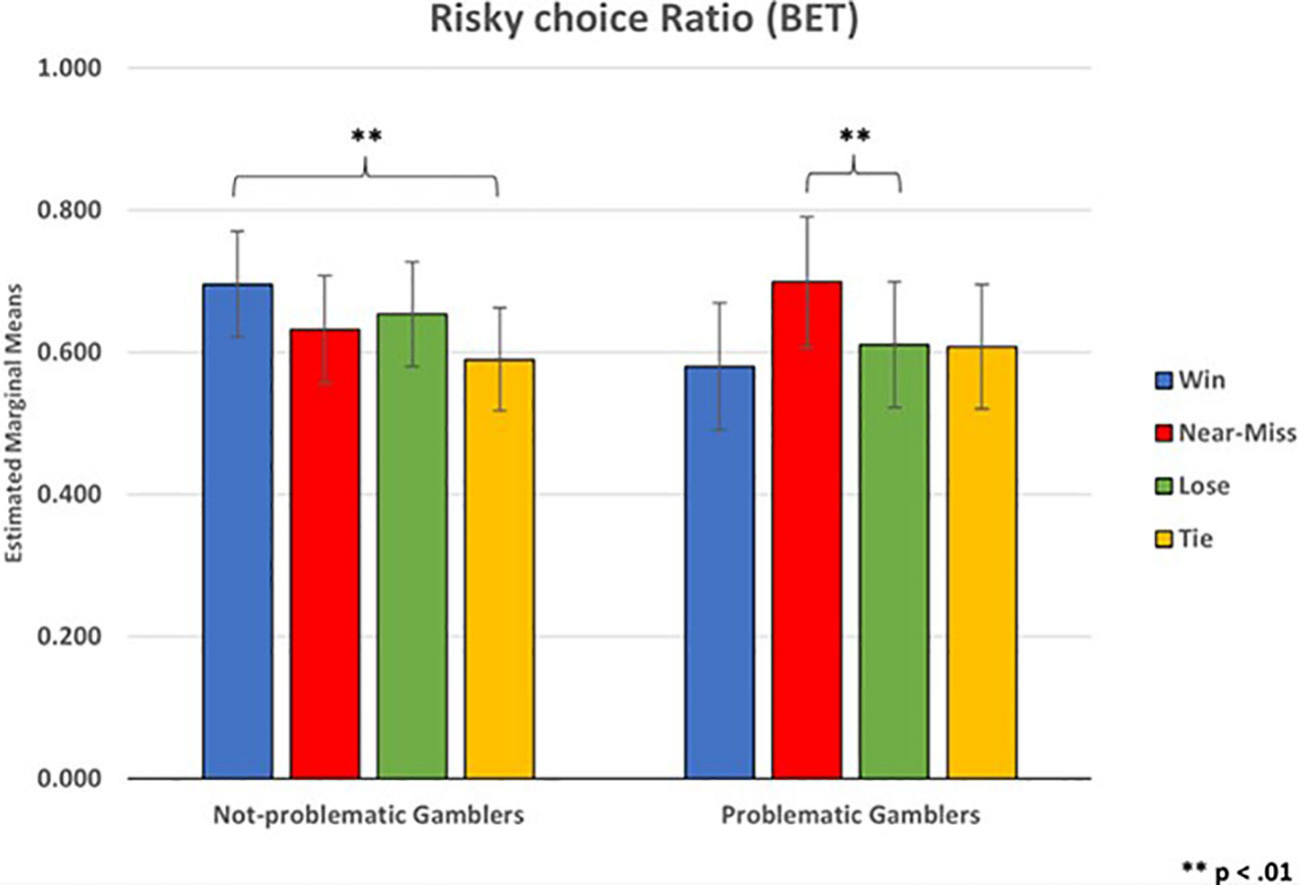
Risky choice ratio (BET).

A 2x4 mixed ANCOVA (group x outcome) was conducted to analyze the Risky bet ratio (i.e., calculated from the bet amount wagered after each outcome). The results showed that the main effect of the outcome was not statistically significant (F_(2.50, 82.63)_ = 1.08, p = .356, ηp2 = .032). Instead, the interaction effect between outcome and group was statistically significant (F_(2.50, 82.63)_ = 4.45, p = .009, ηp2 = .119). Bonferroni corrected pairwise comparisons for the interaction between group and outcome, showed in the not-problematic gamblers group a higher risky bet ratio after a Win (M = .696, SE = .074) than after a Tie (M = .590, SE = .073, p =.033). Also, in problematic gamblers risky bets ratio after a Near-miss (M = .699, SE = .091) was significantly higher than after a Loss (M = .611, SE = .088, p <.009), and higher than after a Win (M = .580, SE = .089) but such a difference was only marginally significant (p = .053) (**Figure 3**). Furthermore, in the ANOVA there was not a statistically significant interaction effect between the Risky bet ratio and the Gaming Addiction covariate (F_(2.50, 82.63)_ = .501, p = .649, ηp2 = .015). There was, instead, a between-subjects effect of the Gaming Addiction covariate (F_(1,33)_ = 6.29, p = .017, ηp2 = .160). Finally, the between-subjects effect of the Group was not -statistically significant (F_(1,33)_ < 1, p = .875).

**Figure 4:**
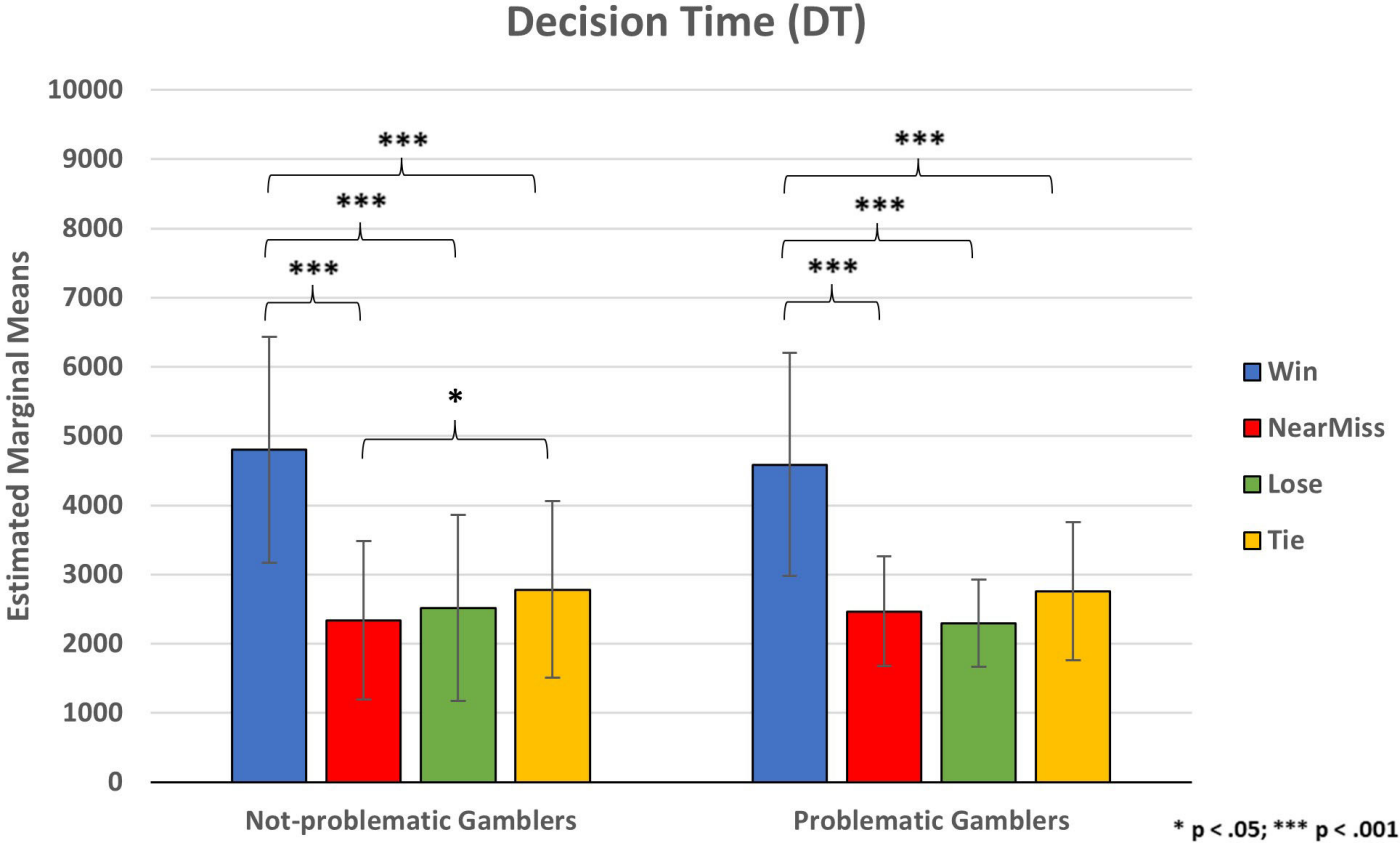
Participants’ decision time for placing bets. * p < .05; *** p < .001.

With respect to the Decision Times (DT), there was a statistically significant main effect of Outcome (F_(1.47, 49.91)_ = 67.64, p <.001, ηp2 = .665), but the interaction between Outcome and Group was not significant (F_(1.47, 49.91)_ = .393, p = .613, ηp2 = .011). Also, there were no significant between-subjects effects of the Group (F_(1, 34)_ < 1, p =.822). Bonferroni corrected pairwise comparisons among the Outcome conditions showed higher decision times after a Win (M = 4695.40, SE = 274.81) than after a Near-miss (M = 2402.38 SE = 171.07, p <.001), a Lose (M = 2405.85, SE = 186.88, p <.001), and a Tie (M = 2771.13, SE = 197.28, p <.001). Such a result is in line with the known Post Reinforcement Pause (PRP) effect (e.g., (12)). Also, after a Tie participants showed higher decision times compared to after Near-misses (p = .01) and Lose (p = .01) (**Figure 4**). Therefore, participants tended to bet earlier after losing points compared to a Tie control condition, where no points are lost.

### 4.2 Physiological results

Regarding HRV, the ANOVA did not reveal a statistically significant main effect of Outcome (F_(2.37, 75.89)_ = 1.82, p = .163, ηp2 = .054), or interaction effect between Outcome and Group (F_(2.37, 75.89)_ < 1, p = .429); there was a statistically significant main effect of IBI (F_(3.00, 96.16)_ = 19.35, p <.001, ηp2 = .377), but not statistically significant interactions between IBI and Group (F_(3.00, 96.16)_ < 1, p = .798), between Outcome and IBI (F_(5.42, 173.58)_ = 1.07, p = .376, ηp2 = .033), and among Outcome, IBI, and Group (F_(5.42, 173.58)_ < 1, p = .568). The between-subjects effect of Group was also not significant (F _(1,32)_ < 1, p = .824).

**Figure 5:**
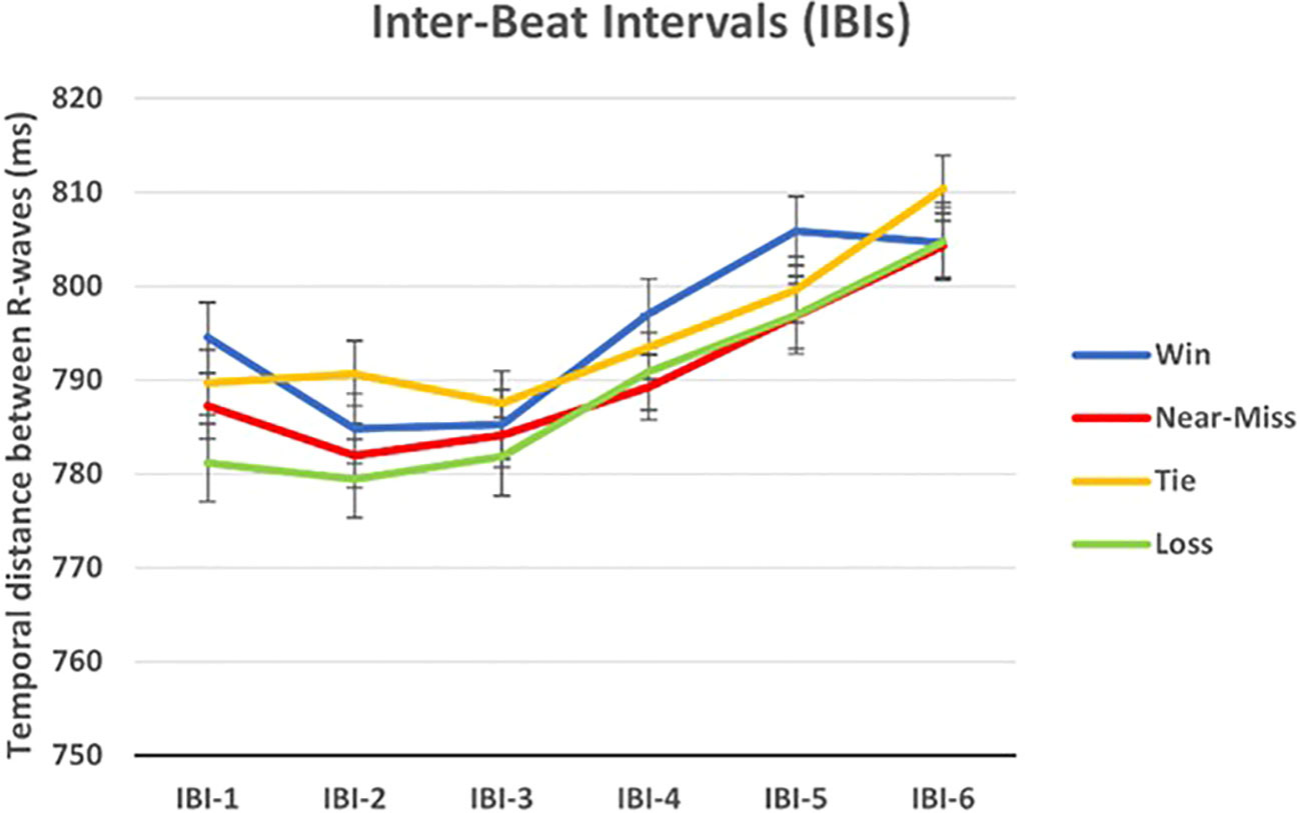
Inter-beat intervals (IBI) for each slot-machine outcome.

Regardless of the type of outcome, a heart rate gradual deceleration appears to occur, peaking just before the last reel stopped (at IBI-5 and IBI-6). Bonferroni corrected pairwise comparisons for the main effect of IBI showed that IBI-6 (M = 806.03, SE = 24.53) was significantly higher compared to IBI-5 (M = 799.80, SE = 24.40, p <.05), IBI-4 (M = 792.70, SE = 24.36, p <.01), IBI-3 (M = 784.67, SE = 24.17, p <.001), IBI-2 (M = 784.23, SE = 25.22, p <.001), IBI-1 (M = 788.15, SE = 25.13, p <.001); while the IBI-5 was significantly higher than IBI-3 (p <.001), IBI-2 (p <.001) IBI-1 (p <.05), and IBI-4 was significantly higher than IBI-3 (p <.05) (**Figure 5**).

With respect to the GSR the ANOVA results showed a statistically significant main effect of Outcome (F_(2.06, 66.08)_ = 3.75, p = .027, ηp2 = .105, there was not a statistically significant interaction effect between Outcome and Group (F_(2.06, 66.08)_ < 1, p = .586), and there was not a between-subjects effect of Group (F_(1,32)_ < 1, p = .690). Pairwise comparisons showed a marginally significant lower mean SCR magnitude in losses (M = 0.102, SE = 0.016) than both ties (M = 0.132, SE = 0.019, p = .053) and wins (M = 0.134, SE = .021, p = .082). Instead, near-misses (M = 0.108, SE = 0.017) were not significantly different from all the other outcome conditions (p >.05).

### 4.3 Regression analyses

A stepwise regression was performed to identify the predictors of SOGS-RA score. At the first step, GRCS-I_Tot (I) was included, which explained the 34.5% of the variability (F_(1,35)_ = 17.88; p <.001). In the second step, Risky choices after Near-Miss ratio (II) was included, which produced a significant increase in the percentage of variance explained 44.3% (F_(2,35)_ = 13.13, p <.001). In the third step, Risky choices after Win ratio (III) was included, explaining the 51.6% of the variance explained (F_(3,35)_ = 11.39; p <.001). In the fourth step, DASS-21_Tot (IV) produced a significant increase in the percentage of variance explained 57.5% (F_(4,35)_ = 10.47, p <.001). Lastly, in the fifth step, DERS-20_Tot (V) produced a significant increase in the percentage of variance explained 63.1% (F_(5,35)_ = 10.25, p <.001). Regression coefficient analysis was reported in **Table 2**.

**Table 2:** Regression coefficients for predicting SOGS-RA.

Model	Predictors	β	t	p	R^2^	R^2^ change	F	p	VIF	p Boot	95%CI boot
SOGS-RA					.631	.569	10.25	p <.001			
	(Constant)	-2.520	-2.191	.036					–	.054	-4.766; -0.153
	GRCS-I_Tot	.055	5.11	.001***					1.05	.004	0.034; 0.088
	Risky_Near-Miss_Ratio	4.50	3.41	.01**					4.34	.003	1.884; 6.854
	Risky_Win_Ratio	-3.02	-2.21	.05*					4.41	.021	-5.229; -0.520
	DASS-21_Tot	-.096	-3.05	.01**					2.1	.029	-0.187; -0.014
	DERS-20_Tot	.054	-2.14	.05*					2.21	.098	-0.006; 0.115

*p <.05; **p <.01, ***p <.001; SOGS-RA, South Oaks Gambling Screen; GRCS-I_Tot, Gambling Related Cognition Scale; DASS-21_Tot, Depressive, Anxiety and Stress Scale; DERS-20_Tot, Difficulties in Emotional Regulation Scale.

## 5 Discussion

This study used a novel VR-powered tool designed to investigate both behavioral and psychophysiological responses elicited by the different kinds of outcomes occurring in a slot machine environment. Despite the current study being preliminary in nature and its main purpose being to test the feasibility and effectiveness of a VR slot-machine environment and to determine whether gambling-related psychophysiological and behavioral responses would be highlighted after a VR exposure, overall, our results seem to be consistent with previous research, supporting both adequate reliability and validity of this VR scenario. Studying gambling in an immersive VR scenario may be a useful tool to evoke erroneous beliefs, especially in problematic gamblers and to highlight the principles underlying the development and maintenance of gambling disorder compared to a low-immersion environment (e.g., a desktop set-up).

Our study also provides new insights, for clarity we divided the discussion into sections, concerning the behavioral, physiological, and clinical results, respectively.

### 5.1 Behavioral results

It is well known that engaging in risky behaviors is often indicative of a propensity towards addictive disorders (e.g., 95). Risk-taking has been associated with different psychological (61) and behavioral factors, for instance, it can be influenced by prior outcomes and past experiences in risk-taking and can be correlated with more frequent wins, partial losses that were disguised as wins, bonus game features, and the largest nominal amount won on a single spin (96). In the literature, two frequently explored phenomena related to the effect of prior outcomes are known as the “escalation of commitment” and the “house money” effect. “Escalation of commitment” indicates an inclination to assume more significant risks following a previous loss, as outlined by Staw (97). On the other hand, the “house money” effect, described by Thaler and Johnson (98), occurs when individuals are more inclined to take greater risks after experiencing a gain.

In line with our hypothesis on risky behavior, the number of risky bets of problem gamblers after a near-miss was significantly higher than after a loss. These findings replicated previous studies on the near-miss effect, according to which slot machine near-misses provoke unpleasant events that increase physiological arousal and frustration, but prolong gambling behavior (12, 41, 99). Several studies (100, 101) have suggested that gambling severity plays a crucial role in the propensity for risky betting after near-misses. In general, problem gamblers have been shown to make more risky decisions from previous losses (“busts”) in a computerized version of Blackjack than after non-losses (“no-busts”; 102). Furthermore, it has been found that pathological gamblers showed an increased risk-taking tendency (103, 104), confirmed by a similar pattern in older adolescent problem gamblers (75). Furthermore, in agreement with Ulrich et al., (24), problem gamblers with more severe gambling problems showed different processing of near-miss outcomes than loss outcomes and impaired processing of gambling outcomes as the severity of gambling problems increased.

Gambling severity seems to predict a greater response in the dopaminergic midbrain to near-miss events, but not to winning events, suggesting that near-misses involve reward-related brain circuits in frequent gamblers (23). The combined effects of the reinforcing nature of wins and the aversive nature of losses could explain the development of the near-miss effect in slot machine gambling from a behavioral perspective. The misconception that near-misses are closer to wins than losses may be the result of a stimulus generalization, in which formally similar stimuli could lead to elicit equal or nearly equal responses (105). In behavioral terms, the opposite is shown by stimulus discrimination, which occurs when a participant responds differently to stimuli despite potential formal similarities. Since stimulus generalization seemed to affect the development of the near-miss effect, interventions aimed at encouraging participants to discriminate between wins and near-misses could have great utility in preventing problem gambling.

An interesting result emerged concerning the Gaming addiction covariate (IGDS-9) which showed a significant main effect with the risky bet ratio. Gaming addiction revealed a certain influence on participants’ gambling behavior, showing positive correlations with the risky bet ratio regardless of the previous outcome. Empirical evidence exists linking addiction-related problem behaviors, including videogame/computer and Internet use, and problem gambling (106, 107). In recent years, the video game industry has implemented a process of “*gamblification*” of video games by employing several gambling-like features (e.g., loot boxes) that allow gamers to obtain abilities, items, and customizations that favor them while playing (108). However, the gambling mechanics employed in loot boxes have led many gamers to perceive their game as a form of gambling, and the purchase of loot boxes shows a significant correlation with the severity of problem gambling (109, 110). The introduction of gambling elements into video games has stimulated research into the potential risks associated with purchasing loot boxes, by highlighting how early exposure can lead the individual toward using real money. In addition, adolescents have been identified as being more likely to engage in novel forms of gambling via the Internet (111), and both gambling and gaming disorder seem to increase among older adolescents (112). The study conducted by Mitchell et al., (113) showed that 15 percent of individuals recognized as Internet addicts were also involved in online gambling and video games.

About decision times, results partially confirmed our hypothesis. Participants showed higher decision times after a win than after all the other outcomes (i.e., ties, losses, and near-misses). This result is consistent with the Post Reinforcement Pause (PRP) (114), but we found no differences between near-misses and losses in which we expected a shorter decision time for the near-miss outcomes. Near-misses, as highly frustrating outcomes, would stimulate the reward system, effectively promoting the maintenance of gambling behavior (13). It has been suggested that the pleasure provided by the reward would inhibit the continuous search for further appealing rewards so that higher PRPs would occur for pleasurable outcomes and none or lower PRPs for frustrating outcomes (12).

In addition, according to several studies (115–118) people would tend to start a new betting round more quickly after a loss than after a win. Such behavior might reflect a higher motivational intensity of “payback” suggesting that, as opposed to the prevailing idea that people become more cautious after unfavorable outcomes, losses (and other frustrating outcomes such as near-misses) in the context of potential rewards are experienced as emotional events that could increase impulsivity (119).

### 5.2 Physiological results

In line with previous results (e.g., 41, 120), we observed a cardiovascular deceleration trend starting from the spin initiation and peaking just before the last reel stopped. Heart rate deceleration has indeed been suggested to represent a marker of feedback anticipation and processing. However, differently from previous findings (121, 122), we observed such a trend regardless of the outcome type or the severity of gambling. Similarly, we did not observe group effects on the SCR, but we found that SCRs after losses were lower than after near-misses, consistent with other studies (37).

Taken together, the physiological measures recorded showed to be sensitive to feedback-related processing in the present novel VR task, further supporting its usage as an ecological measure of gambling behavior.

### 5.3 Self-reported measures

A secondary aim of our study was to investigate which of the self-reported measures considered can predict gambling severity. The final model yielded five predictors that explained 63.1% of the variability. Concerning the first two predictors, regression analysis showed convergent results for gambling-related cognitions and risky choices after a near-miss outcome. Gambling-related cognition scale was found to be positively predictive of problem gambling; this result confirmed both previous findings regarding the relationship between gambling expectancies and problem gambling (100, 123). Furthermore, a recent study conducted on a sample of Italian older adolescents, stated that one of the two most powerful predictors of problem gambling is the interpretive bias (124, 125).

A recent literature review (126) questioned the reinforcing nature of the near-miss outcome, according to which the effect would be limited to the reinforcement of certain types of responses (e.g., initial slot selection or the bet amount) or perhaps the near-miss could only have a respondent function, whereby different conditional emotional responses are elicited. Our result seems to be in line with studies suggesting near-miss outcomes as a means to enhance future gambling responses (127), particularly among gambling disorders (23), reflecting a chasing behavior of continuing to gamble even after losses to make up for previous losses.

The presence of specific personality traits seems to play an important role in decision-making and chasing gambling situations. For instance, from the perspective of Reinforcement Sensitivity Theory (RST; 128), the Behavioral Approach System (BAS) and the Behavioral Inhibition System (BIS) may influence risky or safe decisions following rewarding or punishing feedback in gambling tasks. The risky choices after a Win outcome negatively predicted SOGS score probably suggesting a relative strength of the BIS in affecting decision-making gambling tasks after having a winning experience. This result is in line with previous studies reporting that risky decisions, in a series of gambles, were affected by previous outcomes (13, 129), and in which authors specifically stated that gambler’s fallacy seems to reflect a greater tendency to make more risky choices following a loss compared to following a gain. DASS scale negatively predicted the SOGS score; the literature reported an index of 37.4% of gamblers in recovery communities with an anxiety disorder (130), and was correlated with the severity of problem gambling (131), and especially in adolescence where anxiety may contribute to problem gambling (132). Our results seem to be in line with studies of adolescents that report a relatively low percentage of recognizing negative reinforcement motivations (e.g., gambling motivated by anxiety) as drivers of gambling. In contrast, a higher percentage (67%) reports how gambling is motivated by arousal seeking. These results suggest that positive reinforcement motivations may predominate in gambling behavior during adolescence, as it is a period of high sensation seeking (73, 133).

The DERS scale positively predicts the SOGS score in the stepwise linear regression. Studies have demonstrated a robust positive relationship between DERS and problem gambling (e.g., 134). Ruiz De Lara et al. (135) showed that different aspects of emotion dysregulation were positively linked with the severity of gambling issues. Problem gamblers reported fewer emotional coping strategies, reduced emotional clarity, heightened impulse control difficulties, and lower emotional awareness compared to control subjects. They also experienced greater challenges in accepting, managing, and tolerating their emotions. Research consistently indicates that individuals struggling with gambling problems often turn to gambling as a means of dealing with undesired emotions (136). However, after the bootstrapping procedure, the coefficient of the DERS scale was not significant. Future studies should further investigate these results by considering different types of gamblers with different degrees of addiction separately. The presence or absence of a gambling disorder may have partly influenced the final results.

## 6 Conclusions

Our study showed how VR could be usefully applied in the study, assessment, and observation of gambling-related behavior. In this regard, new technologies, particularly immersive VR, have provided new possibilities for filling some methodological gaps by giving an illusion of a fully surrounding, extended, and vivid reality. This would trigger psychological and physical reactions similar to those in real life, thus allowing for a more ecological investigation of problem behavior. In this sense, our VR scenario appears to be a promising tool in enabling real-time assessments within customized virtual environments or contexts that are perceived and experienced as real, with greater potential to increase the ecological validity compared to the laboratory-based assessment as already observed in several studies (137, 138). The *ad hoc* scenario purposely designed for the gambling context proved to be a useful tool for eliciting behavioral and psychophysiological responses typical of real-game situations (i.e., near-misses effect). In addition, the scenario provides evidence in line with previous studies that have used VR (46, 50, 51, 139, 140).

### 6.1 Limitations

Notwithstanding, the limitations of this study reflect the convenience sampling method, with all high-school students participating (i.e., not representative of gamblers); furthermore, in this study, we only considered participants between the ages of 18 and 20, which may limit the generalizability of our results over the entire adolescent period (i.e., 10-19). Although our VR environment is detailed, it lacks some features that could increase gamblers’ sense of presence and are worth exploring. In particular, the inclusion of other virtual features could increase gamblers’ sense of plausibility. Participants played on a simplified four-reel simulated slot-machine design by using virtual currency instead of real currency. However, the introduction of a real currency would raise challenging ethical considerations that would eventually limit the ecological validity of gambling behavior studies. We have already extensively discussed the added value of the VR environment and how it is more representative of real-world conditions than the laboratory; hence, future studies would benefit from a direct comparison with more realistic conditions.

However, a topic that has been largely neglected is the deceptive illusion and persuasiveness of virtual environments that could affect an individual’s behavior and the ability to distinguish reality from virtuality which could particularly affect the clinical population. Finally, side effects of the VR environment, such as cybersickness and discomfort after prolonged use, should be carefully considered, but are rarely reported in such studies.

In our view, it is important to point out that the population investigated in our study consists of older adolescents, on whom few studies have yet been conducted in the gambling field, however, this study offers new insights and breakthroughs in understanding the relationship between older adolescents and problem gambling. In addition, the sample investigated is not clinical in nature, so the effects found should be interpreted carefully. Therefore, taken together, the results of the present study allow for a significant advance in the ecological assessment of problem gambling among older adolescents.

It is crucial to investigate these aspects for the prevention of potential gambling or gaming disorders, as well as to provide effective clinical interventions tailored to individual characteristics, consistent with the specificity of addiction starting as early as school age.

## Data availability statement

The raw data supporting the conclusions of this article will be made available by the authors, without undue reservation.

## Ethics statement

The studies involving humans were approved by Comitato etico per la Ricerca transdisciplinare di Sapienza Università di Roma. The studies were conducted in accordance with the local legislation and institutional requirements. The participants provided their written informed consent to participate in this study.

## Author contributions

AQ: Conceptualization, Data curation, Formal analysis, Investigation, Methodology, Project administration, Resources, Software, Supervision, Validation, Visualization, Writing – original draft, Writing – review & editing. AP: Investigation, Resources, Supervision, Validation, Visualization, Writing – original draft, Writing – review & editing. CC: Investigation, Software, Supervision, Validation, Visualization, Writing – review & editing. GT: Investigation, Supervision, Validation, Visualization, Writing – review & editing. FF: Investigation, Supervision, Validation, Visualization, Writing – review & editing. JB: Investigation, Project administration, Resources, Supervision, Validation, Visualization, Writing – review & editing. EM: Investigation, Project administration, Resources, Supervision, Validation, Visualization, Writing – review & editing. GL: Investigation, Supervision, Validation, Visualization, Writing – review & editing. AG: Funding acquisition, Project administration, Resources, Supervision, Validation, Visualization, Writing – review & editing. PZ: Conceptualization, Data curation, Formal analysis, Investigation, Methodology, Resources, Software, Supervision, Validation, Visualization, Writing – original draft, Writing – review & editing.
