# Supplementary material for: Gambling and virtual reality: unraveling the illusion of near-misses effect
Source: Front Psychiatry. 2024 Feb 1;15:1322631. doi: 10.3389/fpsyt.2024.1322631 (PMC10867214; doi:10.3389/fpsyt.2024.1322631)
Supplement: Supplementary file 1 [file DataSheet_1.docx]

Supplementary Material

# Supplementary Figures and Tables

## Supplementary Figures


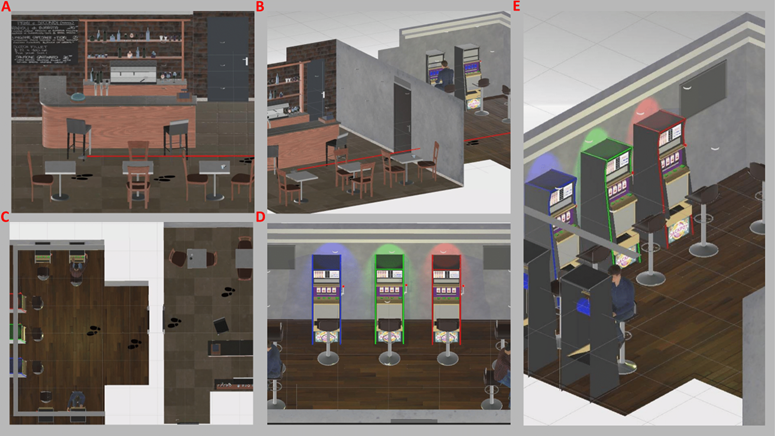


**Supplementary Figure 1.** Virtual Reality Gambling Scenario.


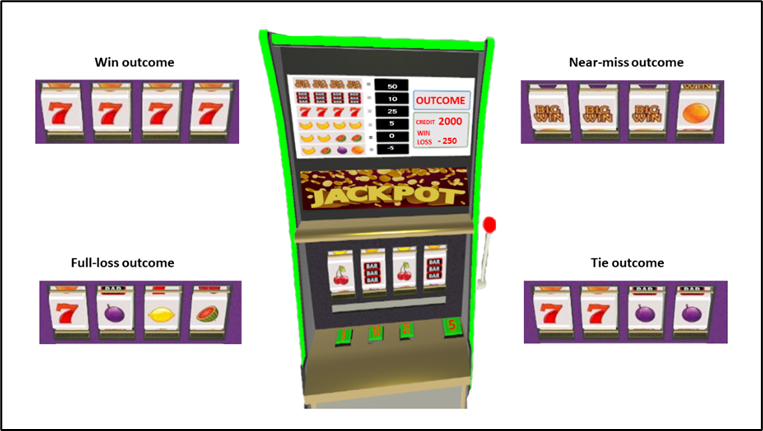


**Supplementary Figure 2.** Slot-machine Outcomes


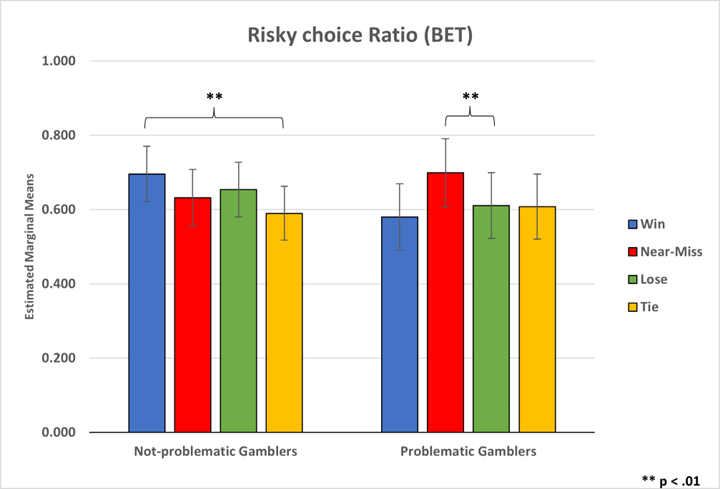


**Supplementary Figure 3.** Risky Choice Ratio (BET)


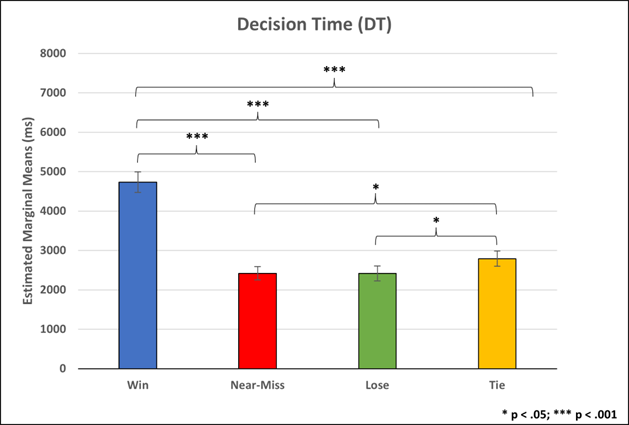


**Supplementary Figure 4.** Participants’ Decision Time for Placing Bets


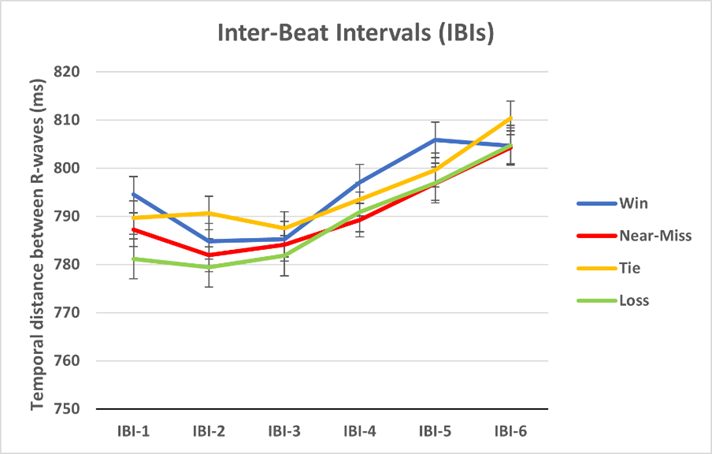


**Supplementary Figure 5.** Inter-Beat Intervals (IBI) for each Slot-machine Outcome
